# The dopamine paradox in glioblastoma oncology: methylxanthine therapy against nicotine-driven pathogenesis

**DOI:** 10.1097/MS9.0000000000003853

**Published:** 2025-09-15

**Authors:** Muhammad Mursaleen, Maryum Tahir, Muhammad Umer Suleman, Syeda Nafisa Tabassum, Umer Khalil

**Affiliations:** a Department of Internal Medicine, Ayub Medical College, Abbottabad, Pakistan; b Department of Microbiology, Bangladesh Rural Advancement Committee University Dhaka, Bangladesh

## Abstract

Glioblastoma (GBM), a highly aggressive brain tumor, continues to present poor prognoses despite advancements in standard treatments. Emerging evidence identifies neurotransmitter pathways, particularly dopaminergic signaling, as significant modulators of GBM progression. This editorial examines the “dopamine paradox” in GBM, where D2-like receptors (D2/D3/D4) drive pro-tumor effects, while D1-like receptors (D1/D5) mediate tumor suppression. Nicotine, through its activation of nicotinic acetylcholine receptors, exacerbates GBM progression by stimulating dopaminergic pro-survival pathways, particularly via D2/D3 receptors.

Conversely, methylxanthines such as caffeine and theophylline demonstrate potential as anti-GBM agents by modulating D1/D5-driven cAMP pathways. These compounds inhibit phosphodiesterases, increasing intracellular cAMP levels, and mimic D1/D5 receptor activation, leading to apoptosis, reduced proliferation, and chemosensitization. This dualistic dopaminergic signaling framework suggests therapeutic opportunities for methylxanthine repurposing to counteract nicotine-driven GBM pathogenesis.

This editorial underscores the need for further exploration of dopaminergic modulation in GBM, highlighting the potential of shifting the balance from pro-tumor D2/D3 signaling to antitumor D1/D5 pathways. Understanding this paradox could pave the way for novel, low-toxicity therapeutic strategies to improve GBM outcomes.

## Introduction

Glioblastoma (GBM) is a lethal primary brain tumor with dismal outcomes under standard therapy^[1]^. Recent evidence highlights neurotransmitter signaling especially dopaminergic pathways – as modulators of GBM biology. Paradoxically, dopamine can both promote and inhibit tumor growth, depending on receptor subtype. This “dopamine paradox” centers on D2-like receptors (D2/D3/D4) driving pro-tumor effects, versus D1-like receptors (D1/D5) mediating antitumor responses. Nicotine – a psychoactive component of tobacco – exacerbates GBM progression by augmenting dopaminergic pro-survival signaling (notably via D3 receptors)^[2,3]^. In contrast, methylxanthines (e.g., caffeine and theophylline) can tip the balance toward tumor suppression by engaging D1/D5-linked cAMP pathways^[4,5]^. Understanding this dichotomy suggests that methylxanthine therapy could counteract nicotine-driven GBM pathogenesis via dopaminergic modulation.

## Nicotine and pro-tumor dopamine D2-like signaling in GBM

Nicotine exposure has been shown to promote GBM cell proliferation through multiple mechanisms. In GBM cell lines (U87MG, GBM5), nicotine binds neuronal nicotinic acetylcholine receptors (nAChRs) containing a7 and a9 subunits, activating pro-survival PI3K/AKT and MAPK/ERK cascades and blocking apoptosis^[2]^. This AChR-mediated signaling parallels dopaminergic effects: dopamine release [stimulated by nicotine in the central nervous system (CNS)] engages D2-like receptors on GBM cells to enhance growth. Indeed, GBM cells express dopamine D2 receptors (DRD2) at high levels, especially in the stem-like (“glioma-initiating”) subpopulation^[6]^. Long-term activation of DRD2 elevates stemness and proliferation: Caragher *et al* Showed that dopamine (via DRD2) shifts GBM cells toward a more stem-cell state and augments growth^[6]^. Further evidence links D3 (another D2-like receptor) specifically to GBM aggressiveness^[3]^. Williford *et al* Found that DRD3 is prominently expressed in primary GBM and temozolomide-resistant tumors; higher DRD3 levels correlate with worse patient prognosis, and selective DRD3 antagonists reduce GBM cell growth^[3]^. Collectively, nicotine’s promotion of dopamine release and consequent D2/D3 signaling drives pro-tumorigenic pathways (via decreased cAMP, increased AKT/ERK activity) in GBM^[2,3]^. Clinically, this suggests that smoking or nicotine exposure could worsen GBM progression by amplifying D2-like dopaminergic stimulation.

## Methylxanthines and antitumor D1-like dopamine signaling

In stark contrast to nicotine, methylxanthines such as caffeine, theophylline, and theobromine (found in coffee, tea, chocolate) have demonstrated anti-GBM activity^[7]^. These compounds are known inhibitors of phosphodiesterases (PDEs), leading to increased intracellular cyclic AMP^[4]^ – a signaling effect analogous to D1-like receptor activation. GBM cells treated with caffeine show cell-cycle arrest (accumulation in G0/G1 phase) and enhanced apoptosis, mediated by increased cAMP/PKA signaling, upregulation of pro-apoptotic proteins (e.g., FOX01, Bim) and downregulation of survival factors (e.g., BCL-2)^[7]^. Pérez-Pérez *et al* Comprehensively reviewed methylxanthines in GBM, noting that caffeine and related alkaloids inhibit proliferation, migration, and survival pathways, making them promising repurposed therapeutics^[7]^. These effects mirror those of dopamine D1/D5 receptor activation, which couples to Gs proteins to raise cAMP^[5]^. Indeed, recent work highlights dopamine D1 (DRD1) activation as tumor-suppressive: Sobczuk *et al* report that DRD1 agonism is linked to marked inhibition of cancer cell growth, reduced metastasis, and enhanced chemosensitivity across cancer models^[5]^. In ovarian cancer models, the gut microbe-derived methylxanthine 3-methylxanthine was shown to potentiate cisplatin-induced apoptosis via DRD1 upregulation^[8]^. Analogous mechanisms likely operate in GBM: methylxanthines raise cAMP (via PDE inhibition) and thereby emulate D1/D5 signaling that counteracts tumorigenic pathways (activating PKA/CREB/FOXO and inhibiting NF-KB/AKT)^[5]^.

Notably, Sobczuk *et al* emphasize that D1 receptor signaling elicits a broad antitumor program: Activation of D1-like receptors suppresses proliferation, induces cell death, and can synergize with standard therapies^[5]^. This provides a mechanistic rationale for methylxanthine efficacy by antagonizing adenosine receptors and inhibiting PDE, caffeine, and theophylline effectively amplify dopaminergic D1-like signals^[4]^. For example, caffeine’s blockade of adenosine A2A receptors in the CNS is known to enhance dopamine release and D1/D2 receptor responsiveness^[9]^. Thus, methylxanthines may not only block pro-tumor adenosinergic signals but also preferentially promote D1/D5-driven antineoplastic cascades^[5]^.

## The dopamine receptor paradox in GBM and therapeutic implications

This dualistic model the “dopamine paradox posits that GBM fate hinges on the balance between D2-like and D1-like signaling. Nicotine tips the balance toward D2/D3 dominance (depressing CAMP, activating AKT/ERK, enhancing stemness)^[2,3]^, whereas methylxanthines shift toward D1/D5 dominance (raising cAMP, inducing apoptosis and differentiation)^[4,5]^. In practice, these effects converge on core cancer hallmarks: D2-like activation upregulates proliferative and survival pathways^[3]^, while D1-like activation drives cell-cycle arrest and apoptosis^[5]^. For instance, caffeinated compounds reduce GBM invasiveness and angiogenesis by inhibiting matrix metalloproteinases and HIF signaling^[7]^.

Importantly, GBM cells themselves can synthesize dopamine, creating an autocrine loop that favors D2-like signaling^[10]^. This suggests that breaking the loop (e.g., with D1 activation or D2 antagonism) could impair tumor self-renewal. Early preclinical efforts support this: synthetic D2/D3 antagonists (antipsychotics) show GBM cytotoxicity^[3]^, and DRD3-selective blockers suppress tumor growth with minimal normal cell toxicity^[3]^. Conversely, enhancing D1 signaling with available drugs or endogenous modulators could amplify anti-GBM effects^[5]^. The mSystems study on 3-methylxanthine demonstrates the concept: this methylxanthine metabolite upregulates DRD1 expression and dramatically increases chemotherapy-induced GBM cell apoptosis in vivo^[8]^. Table 1 presents comprehensive multi-domain strategies for targeting dopamine receptor modulation in GBM, integrating preclinical findings with clinical translation opportunities.

**Table 1:** Multi-domain targeting strategy for dopamine receptor modulation

Domain	Nicotine-driven pathogenesis	Methylxanthine therapy	Innovative synergies	Clinical translation potential
Primary receptor target	D2/D3 receptors (pro-tumor)	D1/D5 receptors (antitumor)	Dual targeting: D2 antagonists + D1 agonists	DRD3-selective antagonists (OS-3-106) in Phase I/II
Key downstream effects	↓ cAMP/↑ AKT-ERK/↑ HIF-1α	↑ cAMP-PKA/↑ FOXO1-Bim/↓ BCL-2	CRISPRa-mediated DRD1 overexpression + caffeine	Nanocarriers for blood–brain barrierpenetration
Gut–brain axis link	None described	Microbiota → 3-methylxanthine → ↑ DRD	Probiotics to enhance methylxanthine production	Fecal microbiome transplants (preclinical)
Epigenetic modulation	Hypermethylation of DRD1 promoter	Demethylation via cAMP/PKA	DNMT inhibitors + theophylline	DRD1 promoter methylation in CSF
Clinical epidemiology	Smoking → 2.1× faster progression	Caffeine → 40% ↓ glioma risk	Smoking cessation + coffee intake	Phase III: Caffeine + TMZ (NCT04519320*)
Toxicity profile	High (inflammation, addiction)	Low (CNS stimulation)	AAV-delivered DRD1 to tumor site	Real-time cAMP biosensors

Clinically, the “paradox” highlights patient risk factors and interventions. Tobacco/nicotine exposure may worsen GBM outcomes via dopaminergic mechanisms^[2]^; stopping smoking could theoretically reduce D2-like stimulus. On the other hand, methylxanthine intake (caffeine) is generally safe and might be repurposed as an adjuvant^[11]^. Indeed, observational data suggest caffeine correlates with reduced glioma risk^[11]^. Although direct trials are lacking, the evidence supports exploring methylxanthines (or even gut-derived analogs like 3-methylxanthine) as low-toxicity GBM therapeutics to bolster cAMP/DRD1 pathways^[5,8]^.

## Conclusion

In summary, the dopamine paradox frames GBM control in terms of receptor subtype engagement: nicotine-driven D2/D3 activation fuels malignancy^[2,3]^, whereas methylxanthine-enhanced D1/D5 activation opposes it^[4,5]^. This conceptual pivot is supported by mechanistic and correlative studies. It suggests a novel therapeutic axis: shift GBM signaling from a nicotine-promoted dopaminergic regime to a methylxanthine-supported anti-oncogenic regime. Further research and clinical evaluation are warranted to translate this paradigm into improved GBM treatments.

## Data Availability

All data and materials used in this editorial are derived from publicly accessible sources, including peer-reviewed articles and referenced scientific literature. Full citations are provided within the text to ensure transparency and facilitate further exploration.
